# Monochorionic triamniotic triplet pregnancy with a co-triplet fetus discordant for congenital cystic adenomatoid malformation of the lung

**DOI:** 10.1186/1742-4755-2-2

**Published:** 2005-04-08

**Authors:** Ahmet Gul, Halil Aslan, Altan Cebeci, Yavuz Ceylan, Ali Ismet Tekirdag

**Affiliations:** 1 Maternal and Fetal Unit, Istanbul Bakirkoy Women and Children Hospital, Istanbul, Turkey; 2 Reproductive Medicine Unit, Istanbul Bakirkoy Women and Children Hospital, Istanbul, Turkey

## Abstract

**Background:**

Spontaneous monochorionic triamniotic pregnancy is rare and is at increased risk for pregnancy complications. The presence of an anomalous fetus further complicates the management.

**Case presentation:**

We present a case of monochorionic triamniotic triplet pregnancy diagnosed at 15 weeks of gestation with one fetus having developed a multicystic lung lesion, suggestive of congenital cystic adenomatoid malformation (CCAM). At 24 weeks, the largest cyst measured 10 mm in diameter. We managed the pregnancy conservatively and delivered three live male fetuses with birth weights 1560 g, 1580 g and 1590 g at 35 weeks of gestation. Two newborns were admitted to the neonatal intensive care unit with respiratory distress, the third one died due to sepsis 7 days postpartum. One of the newborns was discharged healthy at 24 days postpartum. The newborn with CCAM developed a pneumothorax on the right side, recovered after treatment, and was discharged after one month. Computerized tomography (CT) of the infant at 3 months demonstrated two cystic lesions in the middle lobe of the right lung measuring 25 mm and 15 mm. A repeat CT of the infant at 6 months showed a 30 mm solitary cystic mass.

**Conclusion:**

Monochorionic triamniotic triplet pregnancy with a co-triplet fetus discordant for CCAM, present rarely and can be managed conservatively. These findings may help in decision making and counselling of parents.

## Background

The prevalence of spontaneous triplet pregnancy is about 1 in 7000 deliveries, but with the increasing availability of assisted reproductive technologies, the rate of high-order multiple pregnancies has risen dramatically over the last 20 years [1,2]. Although multiple births have increased and most of the reported monochorionic triplet pregnancies have been conceived by in-vitro-fertilisation, the monochorionic triplet pregnancy is rare, and is estimated to be approximately 1 in 100,000 births [3,4].

Triplet pregnancies are at an increased risk for pregnancy complications and have higher perinatal morbidity and mortality rates, such as vascular anastomoses and developmental anomalies. In this report we present a case of monochorionic triamniotic triplet pregnancy with a co-triplet discordant for multicystic lung lesion, suggestive of congenital cystic adenomatoid malformation (CCAM).

## Case presentation

**Figure 1:**
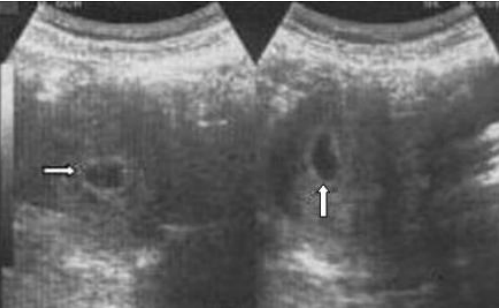
Ultrasonographic image of the case, monochorionic triamniotic triplet pregnancy, is demonstrating single chorionic cavity at 6 weeks of gestation (arrows).

A 26 year-old woman was referred to our maternal and fetal unit for detailed ultrasonographic examination because of triplet pregnancy with threatened abortion at 15 weeks of gestation. Her obstetric history included two first trimester abortions. The patient had taken no medication or drug for ovulation induction. An inquiry into the family history revealed that her mother had delivered triplet babies all of whom died in the early neonatal period. In the present case, the attending obstetrician had performed an ultrasonography at 6 weeks of gestation demonstrating a single, 17 × 20 mm gestational sac (chorion) (Figure 1).

Detailed ultrasonography at our maternal and fetal unit revealed a monochorionic triamniotic triplet pregnancy. Three thin amniotic membranes and an ipsilon zone were detected. The biometric measurements of the three distinct fetuses were appropriate for 15 weeks of gestation. The parents were informed about the risks of a multiple pregnancy and monochorionic placentation. After three days' hospitalization, the vaginal bleeding ceased and the patient was discharged to follow-up.

**Figure 2:**
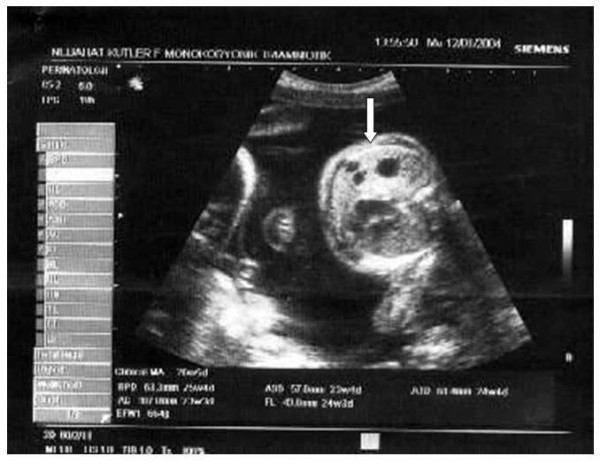
Transabdominal ultrasonography is presenting the co-triplet fetus with multicystic lung lesions, suggesting congenital cystic adenomatoid malformation (arrow) at 24 weeks.

**Figure 3:**
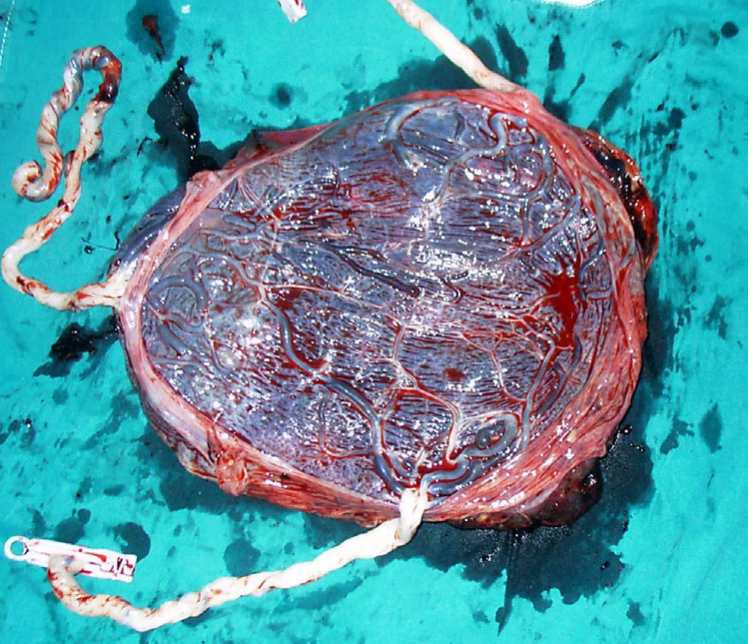
The photograph is demonstrating a single placenta with marginal cord insertion.

The obstetric course was unremarkable until 24 weeks, when ultrasonographic examination revealed that one of the triplet fetuses had developed a multicystic lung lesion suggestive of CCAM, with the largest cyst measuring 10 mm in diameter (Figure 2). Until 30 weeks of gestation, the fetuses had appropriate growth, and follow-up of the pregnancy was uneventful except that the fetus with CCAM developed mild polyhydramnios. At 30 weeks, the patient presented with preterm uterine contractions that ceased after tocolysis with nifedipine 60 mg per day. Betamethasone (12 mg × 2 doses in 24 hours) was administered intramuscularly to the mother to promote fetal lung maturation. The patient was readmitted to our unit at 34 weeks for uterine contractions and impaired fetal growth. The size of the lung lesion remained the same at that time. At 35 weeks of gestation, the patient underwent a low-transverse caesarean section and delivered three live male babies with birth weights 1560 g, 1580 g and 1590 g. Apgar scores were 7/9, 6/8 and 7/9 at 1 and 5 minutes, respectively. A single placenta weighing 1080 g and three distinct membranes were demonstrated (Figure 3). Pathological examination confirmed monochorionic triamniotic placentation. The postnatal course was uneventful and the patient was discharged four days postpartum. Two newborns were admitted to the neonatal intensive care unit for respiratory distress, the third one died due to sepsis on day 7 postpartum. One of the triplets was discharged healthy 42 days postpartum.

**Figure 4:**
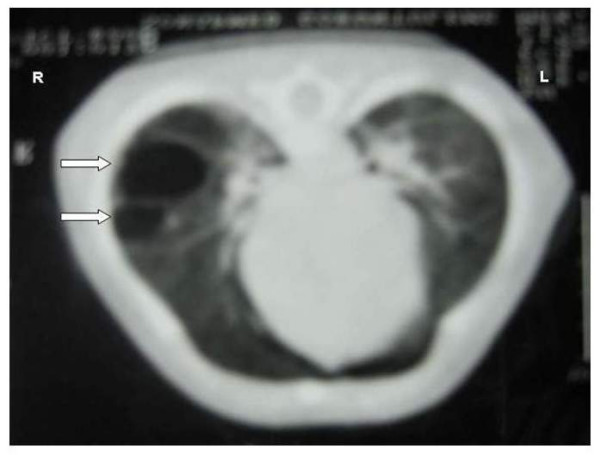
Computerized tomography of the infant at 3 months: two cystic masses in the middle lobe of the right lung, suggesting congenital cystic adenomatoid malformation (arrows).

**Figure 5:**
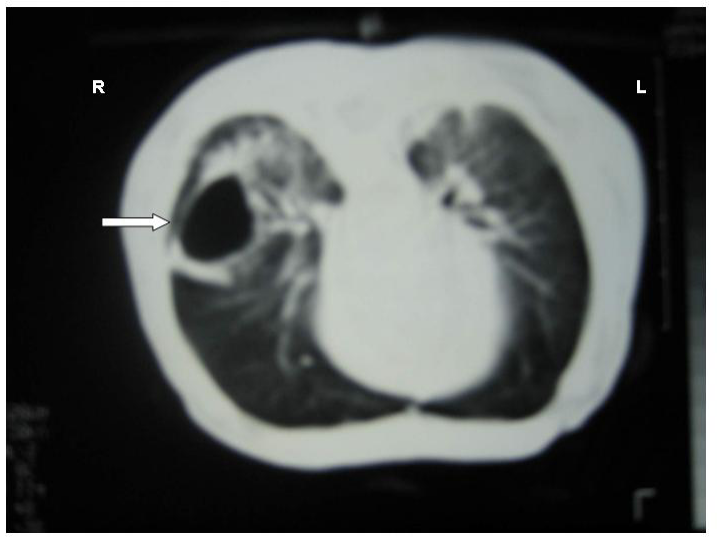
A repeat computerized tomography of the infant at 6 months presents a 30 mm solitary cystic mass (arrow).

The newborn with multicystic lung lesions developed unilateral pneumothorax which was treated by inserting an intercostal drain, and was discharged from the hospital after one month. Computerized tomography (CT) of the infant at 3 months demonstrated two cystic lesions in the middle lobe of the right lung (25 mm and 15 mm in diameter) (Figure 4). A repeat CT of the infant at 6 months showed a 30 mm solitary cystic mass (Figure 5).

## Conclusion

The rate of monozygotic triplet pregnancies in a triplet population is estimated as 4.5%, but the fraction of monochorionic triplets remains unknown [5]. Splitting of the zygote at various stages of development leads to monozygotic multiple pregnancy. The mechanism of monozygotic twinning is not clear. But it is well known that obstetric outcome and clinical management of multiple pregnancies depend on chorionicity. Chorionicity can be established in the first trimester with ultrasound by defining the number of gestational sacs or ipsilon zone [2]. In our case we determined monochorionic placentation by demonstrating a single gestational sac at 6 weeks and ipsilon zone at 15 weeks, and by pathological examination of the placenta after delivery.

The reported incidence of CCAM is approximately one in 10,000–25,000 pregnancies [6,7]. This abnormality is believed to be the result of hamartomatous change in the tertiary bronchioles or an arrest in the embryologic development between 7 and 15 weeks of gestation [8]. It is observed as cystic mass occupying part or the entire fetal lung, predominantly located in the right hemithorax, with up to 15% of cases having bilateral involvement. Prenatal prognostic features for CCAM include size, laterality, progression or regression of the mass, cardiac axis deviation, presentation with hydrops or polyhydramnios [6-8]. Partial or complete regression of the pulmonary lesion is possible. Conservative management is suggested in cases of fetal CCAM without significant mediastinal compression, hydrops fetalis or severe polyhydramnios [6].

Because of the rarity of monochorionic triplet pregnancies, there is no established guideline for management. The presence of an anomalous fetus further complicates the management of pregnancy. Feto-feto-fetal transfusion, acardiac fetus and conjoined twins in triplet gestations have been reported. However, to our knowledge, there are no reports of prenatal diagnosis of a monochorionic triplet pregnancy with a co-triplet fetus discordant for CCAM of the lung.

In our case, a monochorionic triamniotic triplet pregnancy with a co-triplet fetus discordant for CCAM was managed conservatively until 35 weeks of gestation and three live fetuses were delivered. These findings may help in decision-making and counselling of parents.

## Competing interests

The author(s) declare that they have no competing interests.

## Authors' contributions

AG, HA and AC were the consulting perinatologists associated with the case. AG drafted the manuscript, HA participated in the design of the manuscript and AC participated in editing of the manuscript. YC was the director of the Maternal and Fetal Medicine Unit and participated in the design and revision of the manuscript. AIT was the director of the Reproductive Medicine Unit and participated in the design and revision of the manuscript.
